# Coupling control technology of anchoring and unloading in deep intense-mining and large-deformation roadway: a case study

**DOI:** 10.1038/s41598-024-61029-y

**Published:** 2024-05-27

**Authors:** En Wang, Shuaifeng Yin, Qingtao Kang, Xubo Zhao, Qiankun Lan, Hongyuan Sheng, Huiyang Liang

**Affiliations:** 1 School of Mine Safety, North China Institute of Science and Technology, Langfang, 065201 China; 2 Hebei Key Laboratory of Mine Intelligent Unmanned Mining Technology, North China Institute of Science and Technology, Beijing, 101601 China; 3 School of Energy and Mining Engineering, China University of Mining and Technology-Beijing, Beijing, 100083, China

**Keywords:** Civil engineering, Engineering, Geology

## Abstract

In order to control the deformation of surrounding rock in deep high-stress and intense-mining roadways, taking a deep coal roadway with continuous deformation as an example, the characteristics of crustal stress, coal strength, and mining influence of roadway are obtained by underground tests. The combined failure mechanism of coal roadway surrounding-rock is revealed by differential stress of deep and shallow anchor cables. We propose that the improvement of surrounding rock control for coal roadway is adopting the coupling control technology of anchoring and unloading. The stress distribution and evolution laws of lateral surrounding rock of unloading holes are obtained by numerical simulation and theoretical calculation, and reasonable unloading-hole spacing of 4.0 m is comprehensively determined. A mechanical model of roadway roof beam under fixed support at both ends is constructed and the important role of anchor cable beam-truss in controlling the stability of coal roadway is obtained. The rationality of coupling control technology of anchoring and unloading and parameters has been verified by engineering test and mine pressure observation, providing technical references for surrounding rock control in deep intense-mining and large-deformation roadways.

## Introduction

After coal mine enters deep mining, it often leads to large deformation and damage of roadway surrounding-rock, which seriously restricts the safe and efficient mining of deep coal resources^1–3^. The control of large-deformation roadways in deep coal mines has always been a hot research topic. Exploring appropriate control technology in deep high-stress and complex conditions plays an important role in achieving the stability of roadway surrounding-rock^4^.

Deep does not mean that the depth, but a mechanical state, which is jointly determined by surrounding rock properties, mining stress and its crustal stress. It refers to the depth of nonlinear physical and mechanical phenomena exhibited by engineering coal and rock masses and the depth range below it^5–7^. In terms of failure mechanism of surrounding rock in deep mining roadway, Xue et al.^8^ revealed that stress evolution and crack development of roadway surrounding-rock are influenced by weathering and horizontal structural stress, and elucidated the asymmetric distortion characteristic of roadway. Yang et al.^9,10^ analyzed the characteristics of deformation, stress, and crack propagation in deep and soft rock-roadway under deviatoric stress, and elucidated the evolution process of deformation and failure in roadway surrounding-rock. Li et al.^2^ analyzed the main failure characteristic and mechanism of surrounding rock in deep thick-coal-seam roadway, revealing that the central roof is key control point of deformation and failure. Sun et al.^11^ explored the micro mechanism of roadway failure, elucidated the relationship between deformation, rock dip angle, and the position of weak rock masses, and revealed the mechanism of deformation and failure in soft rock-roadway. Sahoo and Palei^12^ come up with a novel control technology for roadway surrounding-rock by adopting risk-based maintenance. Scholars have also used deviatoric stress^13–16^ to study the deformation and failure mechanism of coal roadway surrounding-rock. In terms of unloading and control technology for deep large-deformation roadways, Zang et al.^17^ explored the layered failure characteristic of roadway from shallow to deep, and proposed a combined support technology of "bolt table mesh steel ladder" for deep roadway. Zhao et al.^18^ proposed a support technology of shotcreting, grouting anchor bolt, anchor bolt, and grouting anchor cable for deep soft rock-roadway. Yao et al.^19^ proposed a regional support technology for controlling large deformation of roadway passing through a fault. Scholars have put forward a variety of unloading technical measures such as borehole unloading^20,21^, roof cutting^22–24^, blasting^25^, hydraulic fracturing^26^, as well as asymmetric differential support technology^27–32^ to resist large deformation of coal roadway surrounding-rock under complex conditions of deep high-stress and maintain the stability of surrounding rock in deep roadway.

Given the high stress and complex conditions in deep coal mines, it is difficult to control the deformation of surrounding rock in large-deformation roadways, and it is arduous to effectively control the roadway displacement using a single strengthening support or grouting modification technology. Based on the test of crustal stress and coal strength in deep-mining coal roadway, combined with the differential stress characteristics of deep and shallow anchor cables, this study reveals the deformation and failure mechanism of surrounding rock migration of coal roadway, and puts forward a coupling control technology of anchoring and unloading for coal roadway surrounding-rock. The reasonable spacing of internal unloading-holes of coal roadway is comprehensively determined by numerical simulation and theoretical calculation. A mechanical model of roadway roof beam under fixed support at both ends is constructed, and the important role of anchor cable beam-truss structure in maintaining the stability of coal roadway is obtained. Reasonable coupling control parameters of anchoring and unloading are proposed. The important role of coupling control technology in maintaining the stability of deep-mining coal roadway has been verified by engineering test and mine pressure observation, providing technical references for the control of complex and large-deformation surrounding rock in deep-mining roadways.

## Mine pressure characteristics in mining roadway

Taking the Dongpang Coal Mine of Jizhong Energy Co., Ltd., China as engineering project, this section introduces the engineering overview, deformation and failure characteristics, control difficulties, and mining characteristics of coal roadway. It further reveals the mechanism of continuous deformation and failure, and points out the improvement strategy of surrounding rock control in coal roadway.

### Engineering overview and failure characteristics

The total thickness of No. 2 coal seam in Dongpang Coal Mine is 4.60–6.50 m, with an average of 5.40 m. The average inclination angle of the coal seam is 5°, and the average burial depth is 660 m. The direct roof is 2.32 m siltstone, the basic roof is 8.90 m fine sandstone, and the direct floor is 1.06 m fine sandstone. The chamber of centralized liquid supply pumping station of No. 12 mining area in No. 2 coal seam is a coal roadway with 5.0 m width and 3.0 m height. It is arranged along the roof of the coal seam and is 75 m away from the main track roadway in No.12 mining area and the design final-mining-line of 21,215 coal face. The coal around the roadway exhibits typical characteristics such as looseness and fragmentation, leading to significant deformation throughout the year. Expansion and repair are required every six months to a year, making it difficult to achieve long-term effective control of large-deformation coal roadway.

**Figure 1:**
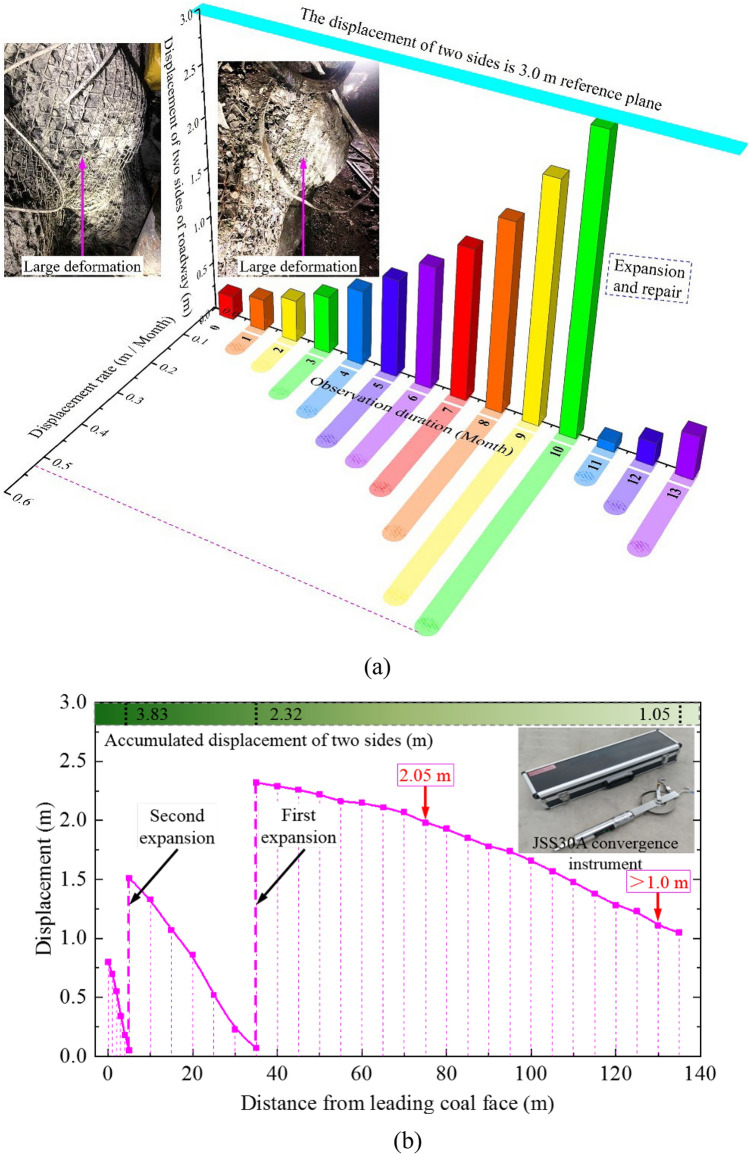
Observation results of roadway deformation around 21,215 coal face. (**a**) Deformation of roadway surrounding-rock without disturbance of coal face, (**b**) Roadway displacement at different positions ahead of the coal face.

When not affected by the disturbance of 21,215 high-mining coal face, two ribs’ surrounding rock of coal roadway undergoes continuous deformation and damage all year round. A special construction team needs to be arranged to continuously expand and renovate it (Fig. 1a) to maintain the basic operation of the roadway, resulting in high support costs. During the mining process of 21,215 high-mining coal face, the carriage roadway located at the same level between the designed final-mining-line of the coal face and coal roadway has been completely closed. The observation results of surrounding rock displacement in two ribs of haulage roadway in the front section of the coal face are shown in Fig. 1b. During the mining process of 21,215 high-mining coal face, the displacement of both ribs within the range of 130 m in front of the coal face exceeds 1.0 m, and the deformation of both ribs at 75 m in front of the coal face exceeds 2.0 m. Therefore, due to the severe disturbance of 21,215 high-mining coal face, the mine pressure in roadway surrounding-rock beyond the front section of the coal face exhibits severe disturbance, and the mining influence range on surrounding rock far exceeds 130 m.

### Crustal stress and coal strength test of roadway

Mastering the stress environment of roadway surrounding-rock is necessary for analyzing its physical and mechanical properties of surrounding rock and simulating roadway stability. Therefore, two aspects of study on physical and mechanical characteristics of coal roadway are carried out: crustal stress test for deep coal roadway and physical and mechanical test for laboratory coal. It can provide measured data support for analyzing large deformation and failure mechanisms of coal roadway.

#### Crustal stress test

The crustal stress of surrounding rock near the coal roadway is measured by stress relief hollow inclusion method. Three typical measuring stations are selected near the coal roadway and hollow inclusions are arranged for crustal stress test. The drilling depth of each measuring point is 12 m, and the stress relief depth is 320 mm. The maximum horizontal principal stress around the coal roadway is 22.18 MPa, the minimum horizontal principal stress is 11.45 MPa, and the vertical stress is 16.72 MPa after averaging the crustal stress.

#### Coal strength test

The joints and fractures in No.2 coal seam of Dongpang Mine are extremely developed, and the coal structure is loose and fragmented. Almost all the original structure has been destroyed, showing significant characteristics of loose, soft, and fragmented in deep coal mines. The mechanical strength of 9 sets of samples is tested in the laboratory, and the results show that the maximum uniaxial compressive strength of the coal around the roadway is 8.763 MPa, the minimum is 2.632 MPa, and the average strength is 5.405 MPa. Therefore, the ratios of average coal strength around the roadway to three principal stresses mentioned above are 0.24, 0.47, and 0.32, respectively. The minimum principal stress has exceeded the compressive strength of coal roadway (up to 2.12 times), and the maximum principal stress is 4.10 times of uniaxial compressive coal-strength. Therefore, the coal roadway is prone to plasticization and destruction under deep and high ground stress.

### Mining characteristics of coal roadway

**Figure 2:**
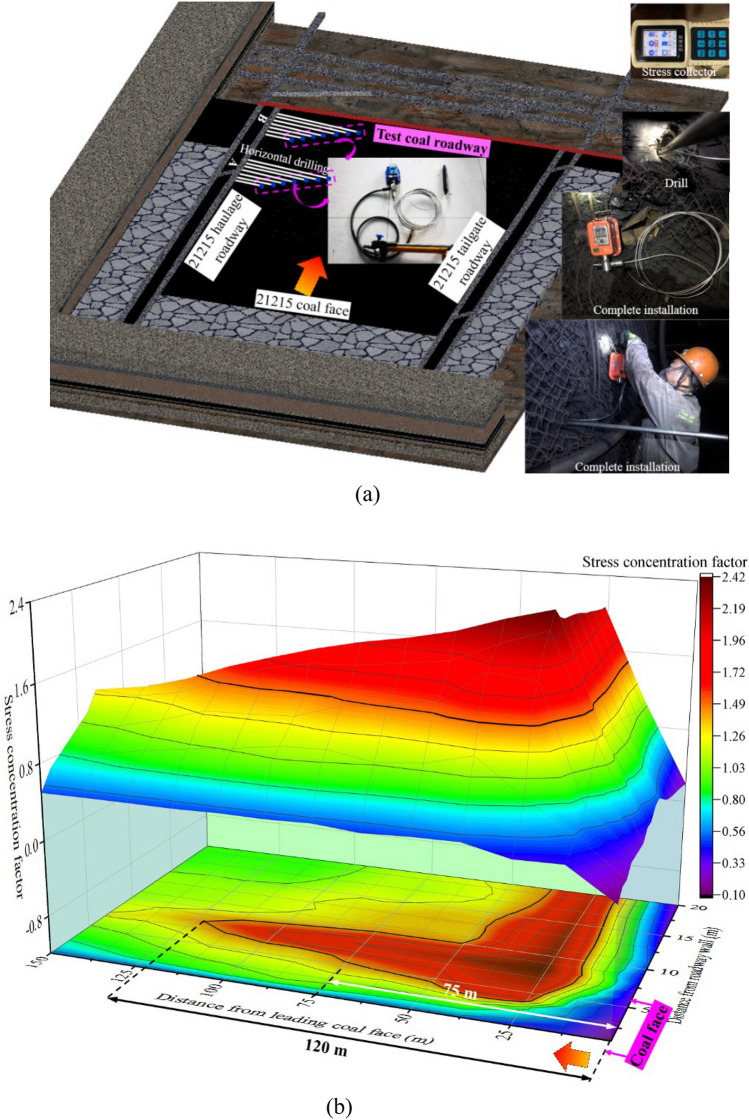
Observation and distribution in advance section of surrounding rock stress around 21,215 coal face. (**a**) Testing plan and process for borehole stress, (**b**) Spatial distribution of surrounding rock stress in advance section of the coal face.

The borehole stress test is adopted to dynamically monitor the mining stress of roadway surrounding-rock in mining process. Each measuring station is equipped with different numbers and depths of borehole stress meters (Fig. 2a), and three-dimensional spatial stress of surrounding rock converted into the mining-stress concentration coefficient is shown in Fig. 2b. The stress concentration factor is equal to the ratio of on-site measured stress to original borehole stress.

There are two peak-stress concentration areas in surrounding rock of the front section of 21,215 coal face: (1) the peak zone of abutment pressure in front of the coal wall parallel to the coal face, about 26 m; (2) the peak zone of abutment pressure perpendicular to the coal face and parallel to the axial direction of the roadway. When the mining face reaches 150 m away from the measuring station, the roadway enters the disturbance influence-area; and when the mining face reaches about 120 m away from the measuring station, the roadway has obviously entered the mining influence-area. When the coal face is 75 m away from the measuring station, the stress concentration coefficient of the roadway is more than 1.60, indicating that surrounding rock at the position 75 m in front of the coal face is strongly influenced by the mining of large-mining-height coal face. Therefore, when the coal face is mined to the design final-mining-line (75 m away from the coal roadway), a larger range of coal roadway damage will occur under the intense-mining influence of the coal face with large-mining height. The intense-mining influence is an important characteristic of coal roadway at present.

### Failure mechanism of coal roadway distortion

**Figure 3:**
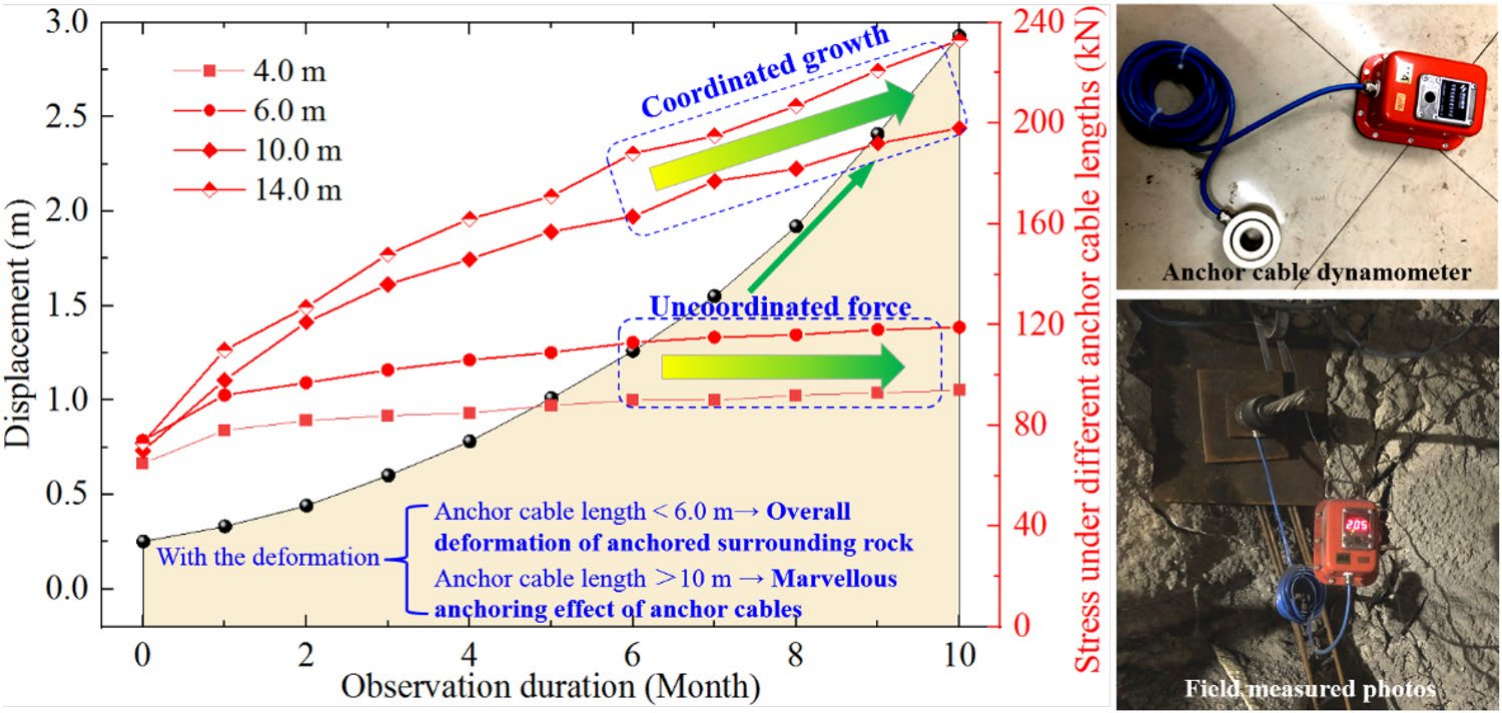
Stress curves of anchor cables with different lengths in the deformation process of roadway.

In order to explore the deformation and failure mechanisms of continuous large-deformation of two ribs in deep coal roadway, the dynamic observation of anchor cables stress in different lengths during continuous deformation was carried out by field test. The observation scheme is as follows: the anchor cables of the same height and different lengths (4.0–14.0 m) are arranged in two ribs of coal roadway, and the stress variation laws of anchor cables during surrounding rock deformation are continuously monitored. The results are shown in Fig. 3.

In the process of continuous deformation of two ribs in coal roadway, the stress variation laws of anchor cables with different lengths are completely different. (1) When anchor cable lengths are 4.0 m and 6.0 m, the stresses are not coordinated with surrounding rock deformation, that is, anchor cable stress maintains a relatively stable state in the process of continuous increase of surrounding rock deformation, which is obviously not synchronous and inconsistent with the mine pressure of roadway surrounding-rock. (2) When anchor cable length increases to 10.0 m, the stress also increases to a certain extent with continuous deformation process of surrounding rock. The anchor cables can anchor the surrounding rock and limit the deformation of coal roadway. (3) As anchor cable length increases to 14.0 m, the stress changes greatly compared with that when anchor cable length is 10.0 m, and the maximum stress can reach 238 kN, showing the coordinated characteristic consistent with the deformation trend of two ribs’ surrounding rock of coal roadway. It is concluded that anchor cable with length of 14 m plays a marvelous anchoring role in roadway surrounding-rock significantly.

In summary, when anchor cable length is small (< 6 m), the damage range of surrounding rock in both ribs of coal roadway exceeds the scope of anchor cable support. The surrounding rock in shallow anchoring rock of two ribs will be squeezed out as a whole by deep coal body, which means that shallow anchoring rock will undergo overall structural extrusion deformation. When anchor cable length is greater than 10.0 m, it can play a certain anchoring role, and when anchor cable length increases to 14.0 m, the anchoring effect is better. It can be concluded that the failure depth of surrounding rock in two ribs of coal roadway is large (beyond the support range of anchor cables), and the whole surrounding rock of shallow anchorage supporting structure is extruded, which leads to continuous large-deformation of surrounding rock. The joint failure mechanism of large deformation is revealed and formed, in which entire coal mass in the deep area migrates into the coal roadway and acts on the anchoring support system.

### Improvement strategy for coal roadway control

**Figure 4:**
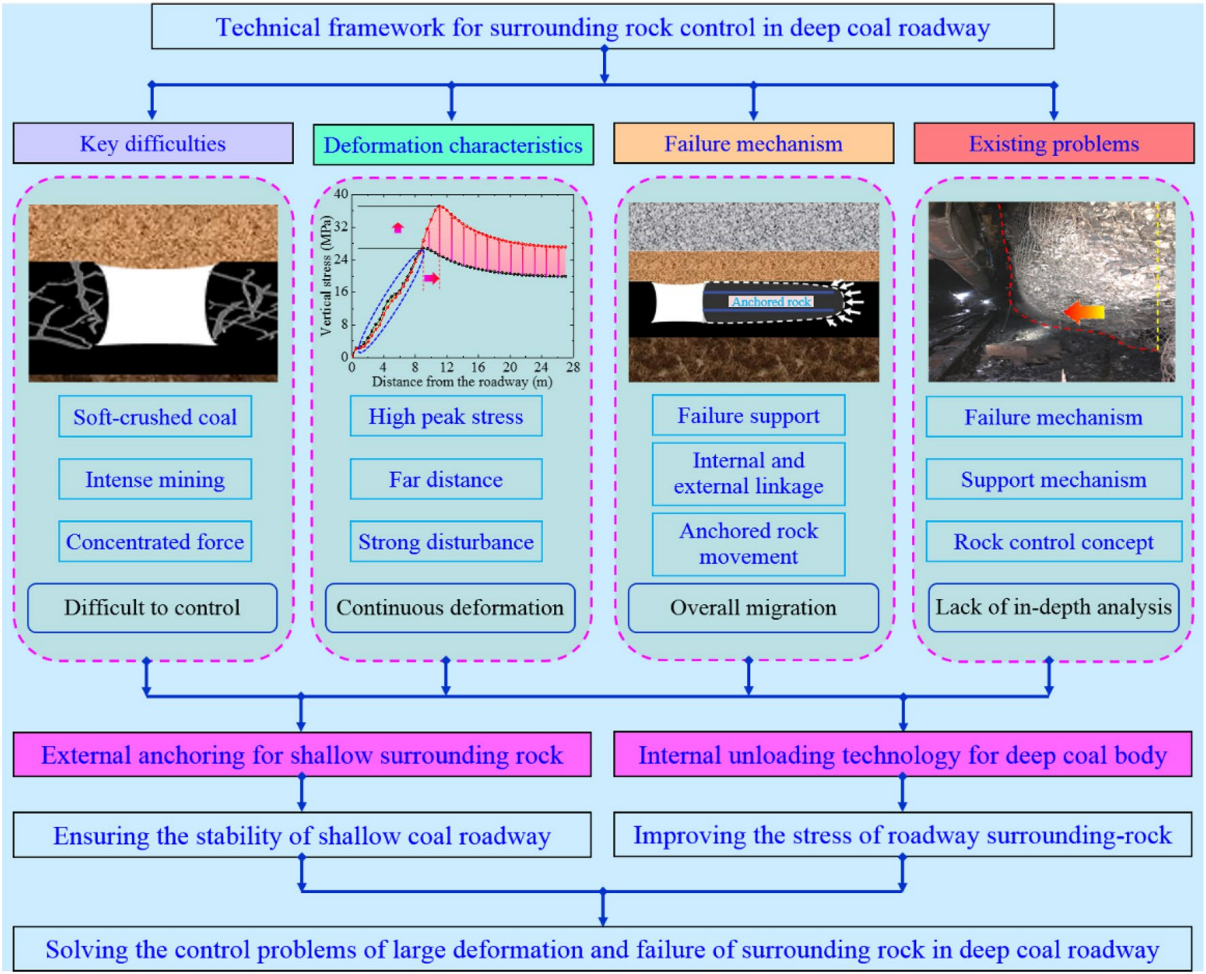
Technical framework of surrounding rock control in deep coal roadway.

The surrounding rock control of deep coal roadway in Dongpang Mine exists the following problems: (1) unclear understanding of deformation and failure mechanism; (2) unclear understanding of the interaction between surrounding rock and support; (3) the control idea of surrounding rock is not suitable. Based on the failure mechanism of continuous movement of coal in the deep area of two ribs, which acts on the anchoring surrounding rock to cause overall deformation, it is analyzed that only conventional strengthening support or grouting modification technology is no longer suitable for roadway surrounding-rock control. Starting from the essence and mechanism of continuous deformation and failure of surrounding rock in intense-mining roadway, this study proposes a coupling control technology of anchoring and unloading that can improve the stress environment of roadway surrounding-rock. The technical framework is shown in Fig. 4.

In the coupling control technology of anchoring and unloading for deep coal-roadway surrounding-rock, anchoring refers to the comprehensive control measures such as strengthening support with anchor cable beam-truss and grouting modification in the shallow of coal roadway to improve the strength and bearing capacity of coal roadway surrounding-rock. And unloading refers to the implementation of cave-making and pressure relief technology in two ribs of coal roadway to transfer the high concentrated-stress to the deep, thereby improving the overall stress environment of coal roadway surrounding-rock.

## Determination of internal unloading-hole spacing of coal roadway

Based on the determination of reasonable internal unloading-hole position, unloading-hole length and other key technical parameters, this section mainly analyzes the three-level classification and standard of unloading-hole position on stability of coal roadway, and comprehensively determines reasonable spacing of internal unloading-holes by theoretical calculation and numerical simulation.

### Basic parameters of internal unloading for coal roadway surrounding-rock

In this section, a three-dimensional numerical model of FLAC for surrounding rock of deep coal roadway in Dongpan Mine is established, which is based on the Hoek–Brown criterion for processing laboratory rock mechanics and numerical simulation mechanics parameters^33^. The load on the upper model is 16.50 MPa, and the lateral pressure coefficient is 1.2. The Mohr–Coulomb model is used as the constitutive model of coal roadway deformation and failure. Based on the existing research^34,35^, the reasonable internal unloading parameters are determined as follows: (1) the unloading-hole position is 10.0 m away from the roadway wall; (2) the unloading-hole length is 5.0 m. This section mainly studies the response laws of unloading-hole spacing to the stability of coal roadway and determines reasonable unloading-hole spacing.

**Figure 5:**
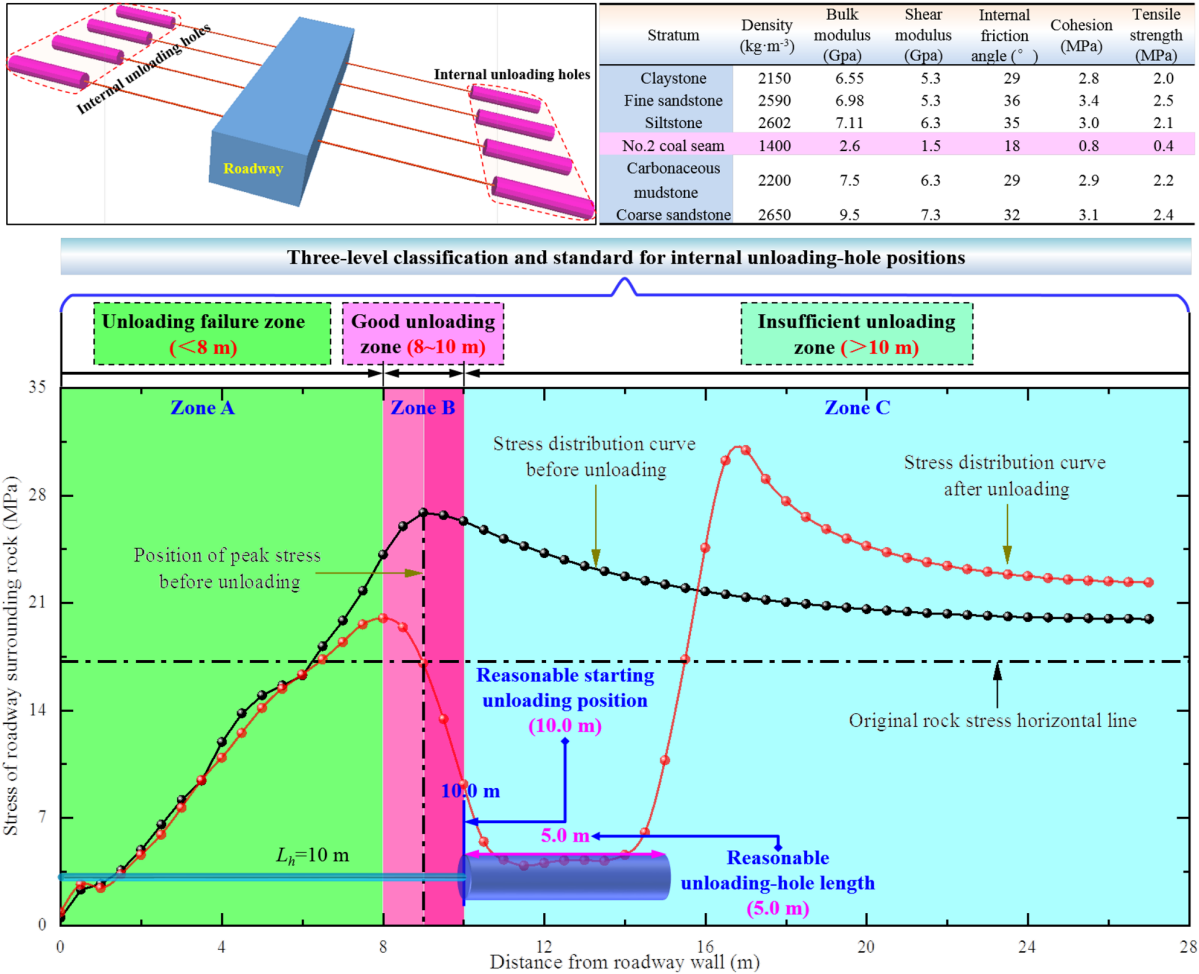
Numerical model and three-level division of different internal unloading-hole positions.

Based on the stress distribution, expansion, and evolution laws of surrounding rock under different internal unloading-hole positions and lengths in two ribs of coal roadway by numerical simulation, a three-level classification and standard for different unloading-hole positions are obtained (Fig. 5). (1) Good unloading zone: located within the peak-stress zone within 2.0 m of two ribs of coal roadway (the original peak position extends 1.0 m inward and 1.0 m outwards, forming Zone B), especially when unloading is carried out outside the original peak-stress line (near solid coal side), it can significantly transfer the original high-concentrated stress of roadway to a deeper depth, without affecting the stability of roadway surrounding-rock in shallow anchoring support. (2) Unloading failure zone: as unloading position is in the inner side of peak-stress zone (zone A) within the range of 2.0 m, the stress transfer effect of coal roadway surrounding-rock is not obvious, and it is easy to damage the integrity of shallow anchoring surrounding-rock of coal roadway, which is easy to induce the loss of bearing capacity of surrounding rock in two-rib anchoring area. (3) Insufficient unloading zone: as unloading position of two ribs is outside the peak-stress zone (zone C) within the range of 2.0 m, the magnitude and location of internal peak-stress are basically consistent with that before unloading, resulting in insufficient unloading of roadway surrounding-rock.

### Numerical analysis of internal unloading-hole spacing

**Figure 6:**
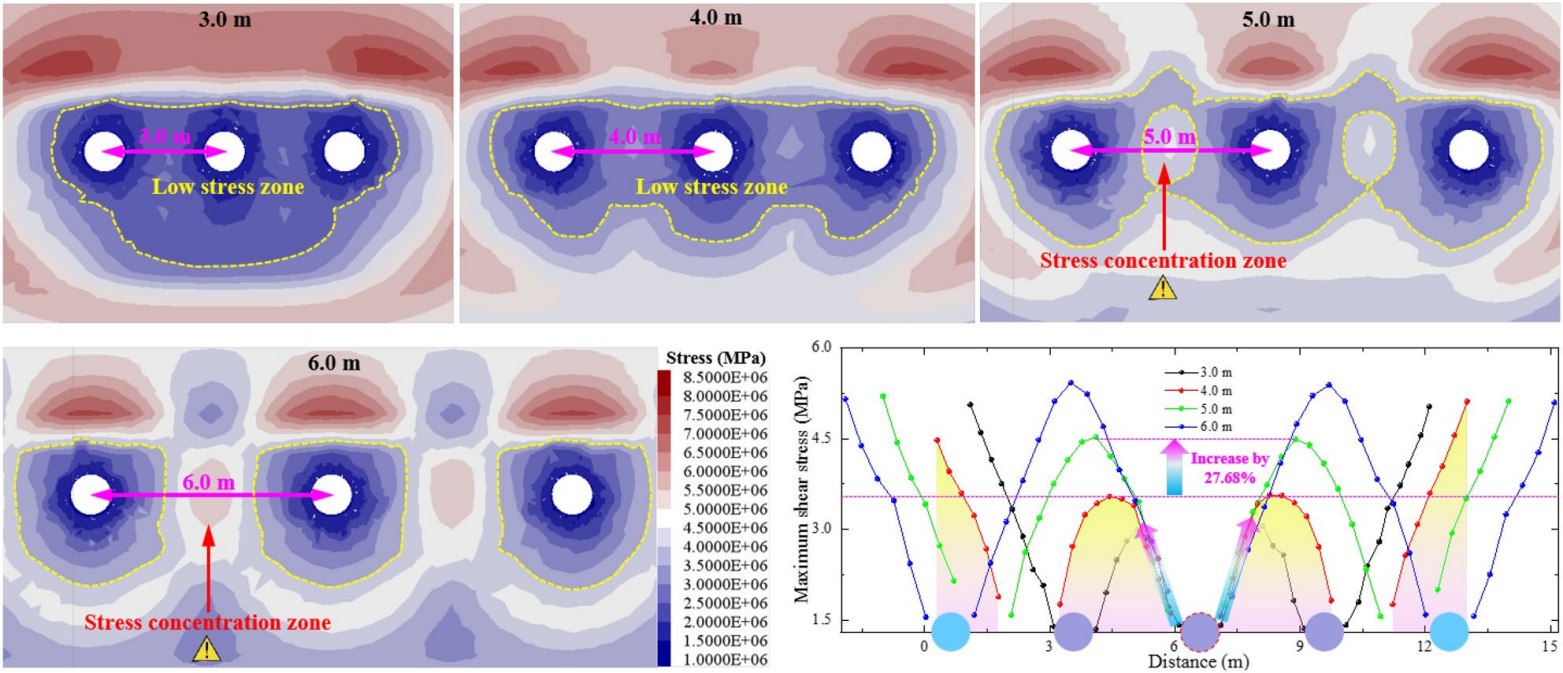
Stress distribution of surrounding rock under different unloading-hole spacing.

Based on determining reasonable internal unloading-hole position and length, the maximum shear stress of roadway surrounding-rock under different unloading-hole spacing is studied by numerical simulation, as shown in Fig. 6. When unloading-hole spacing is less than 4.0 m, the hole surrounding-rock is in a low-stress state, which can ensure that anchoring surrounding rock of coal roadway is not deteriorated on the basis of realizing significant transfer of high concentration stress to the deep. It is conducive to the overall stability of internal unloading-holes and roadway surrounding-rock. As unloading-hole spacing increases to 5.0 m, a new high-concentrated stress zone appears between unloading holes, and the maximum concentrated stress increases by 27.68% compared with the spacing of 4.0 m. The surrounding rock between cavitation holes is inadequate pressure relief. When unloading-hole spacing is larger, the surrounding rock will induce secondary failure under the high stress, which is not conducive to the stability control of coal roadway surrounding-rock. Therefore, internal unloading-hole spacing should be ≤ 4.0 m.

### Theoretical calculation and analysis of internal unloading-hole spacing

**Figure 7:**
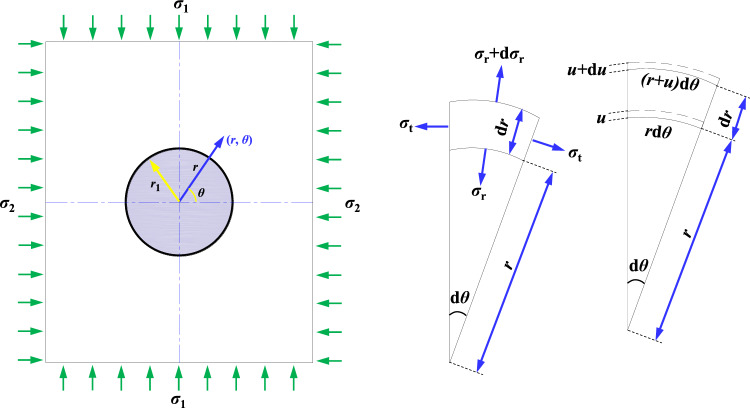
Stress distribution of unit body around internal unloading circular-hole.

Due to the complex boundary conditions of underground roadways in coal mines, which are heterogeneous, discontinuous, and nonlinear geometric bodies, it is currently impossible to accurately solve the stress state of surrounding rock using mathematical and mechanical methods. To simplify the calculation, it is assumed that surrounding rock in coal mine is homogeneous, isotropic, linear elastic, and without creep or viscous characteristic. The original rock stress is in an isotropic and isobaric state, and thus the plane strain problem^36,37^ can be used to solve internal circular unloading-hole spacing. Figure 7 shows the stress distribution of unit body around internal unloading hole, which can be used to list the equilibrium equation of unloading hole as follows:

1 $$(\sigma_{{\text{r}}} + {\text{d}}\sigma_{{\text{r}}} )(r + {\text{d}}r){\text{d}}\theta - \sigma_{{\text{r}}} r{\text{d}}\theta - 2\sigma_{{\text{t}}} {\text{d}}r\sin \frac{{{\text{d}}\theta }}{2} = 0$$

where, *σ*_r_ and *σ*_t_ are the radial stress and tangential stress of unloading-hole surrounding-rock, respectively. *r* and *θ* are the radius and coordinate angle of unit body, respectively.

Considering that the value of $\frac{{{\text{d}}\theta }}{2}$ is very small, it can be approximated as $\sin \frac{{{\text{d}}\theta }}{2} = \frac{{{\text{d}}\theta }}{2}$, then:

2 $$\sigma_{{\text{r}}} - \sigma_{{\text{t}}} { + }r\frac{{{\text{d}}\sigma_{r} }}{{{\text{d}}r}} = 0$$

The geometric equation for surrounding rock in circular unloading-hole is as follows: as radial deformation of unit body is in the process of *u* → *u* + d*u*, the expression of radial strain is:

3 $$\varepsilon_{{\text{r}}} = \frac{{(u + {\text{d}}u) - u}}{{{\text{d}}r}} = \frac{{{\text{d}}u}}{{{\text{d}}r}}$$

The expression of tangential strain *ɛ*_t_ resulting from *r*d*θ* → (*r* + *u*)d*θ* in the course of tangential deformation of unit body is:

4 $$\varepsilon_{{\text{t}}} = \frac{{(r + u){\text{d}}\theta - r{\text{d}}\theta }}{{r{\text{d}}\theta }} = \frac{u}{r}$$

5 $$\frac{{{\text{d}}\varepsilon_{{\text{t}}} }}{{{\text{d}}r}} = \frac{1}{r}\frac{{{\text{d}}u}}{{{\text{d}}r}} - \frac{u}{{r^{2} }} = \frac{1}{r}(\frac{{{\text{d}}u}}{{{\text{d}}r}} - \frac{u}{r}) = \frac{1}{r}(\varepsilon_{{\text{r}}} - \varepsilon_{{\text{t}}} )$$

From the generalized Hooke's law:

6 $$\varepsilon_{{\text{t}}} = \frac{1}{E}\left[ {\sigma_{{\text{t}}} - \mu (\sigma_{{\text{r}}} + \sigma_{{\text{z}}} )} \right]$$

7 $$\varepsilon_{{\text{r}}} = \frac{1}{E}\left[ {\sigma_{{\text{r}}} - \mu (\sigma_{{\text{t}}} + \sigma_{{\text{z}}} )} \right]$$

where, *σ*_z_ is the axial stress of surrounding rock of internal unloading-hole.

8 $$\frac{1}{r}(\varepsilon_{{\text{r}}} - \varepsilon_{{\text{t}}} ) = \frac{1}{r}\frac{1}{E}\left[ {\sigma_{{\text{r}}} - \mu (\sigma_{{\text{t}}} + \sigma_{{\text{z}}} ) - \sigma_{{\text{t}}} + \mu (\sigma_{{\text{r}}} + \sigma_{{\text{z}}} )} \right] = \frac{1 + \mu }{{rE}}(\sigma_{{\text{r}}} - \sigma_{{\text{t}}} )$$

Due to:

9 $$\frac{{{\text{d}}\varepsilon_{{\text{t}}} }}{{{\text{d}}r}} = \frac{1}{E}\left[ {\frac{{{\text{d}}\sigma_{{\text{t}}} }}{{{\text{d}}r}} - \mu \frac{{{\text{d}}\sigma_{{\text{r}}} }}{{{\text{d}}r}}} \right]$$

Available:

10 $$\frac{{{\text{d}}\sigma_{{\text{t}}} }}{{{\text{d}}r}} - \mu \frac{{{\text{d}}\sigma_{{\text{r}}} }}{{{\text{d}}r}} = \frac{1 + \mu }{r}(\sigma_{{\text{r}}} - \sigma_{{\text{t}}} )$$

If the expression (2) and (10) are combined, *σ*_1_ = *γH*, the expression of *σ*_r_ and *σ*_t_ at any point of unloading circular-hole can be expressed as follows:

11 $$\sigma_{{\text{r}}} = \gamma H(1 - \frac{{r_{1}^{2} }}{{r^{2} }})$$

12 $$\sigma_{{\text{t}}} = \gamma H(1 + \frac{{r_{1}^{2} }}{{r^{2} }})$$

where, *r*_1_ is the radius of internal unloading circular-hole.

In Fig. 7 above, the static equilibrium equation of limiting equilibrium zone of surrounding rock of internal unloading circular-hole is:

13 $$r\frac{{{\text{d}}\sigma_{{\text{r}}} }}{{{\text{d}}r}} + \sigma_{{\text{r}}} - \sigma_{{\text{t}}} = 0$$

The expression of limit equilibrium condition is:

14 $$\sigma_{{\text{t}}} = \frac{1 + \sin \varphi }{{1 - \sin \varphi }}\sigma_{{\text{r}}} + \frac{2C\cos \varphi }{{1 - \sin \varphi }}$$

where, *σ*_r_ and *σ*_t_ are the radial stress and tangential stress of surrounding rock of unloading hole in limit equilibrium zone, respectively. *C* and *φ* are the cohesion and internal friction angle of coal around unloading holes, respectively. *r* is the radius of surrounding rock corresponding to a point in limit equilibrium zone.

By substituting Eq. (14) into Eq. (13), we can get:

15 $$r\frac{{{\text{d}}\sigma_{{\text{r}}} }}{{{\text{d}}r}} + \sigma_{{\text{r}}} - \frac{1 + \sin \varphi }{{1 - \sin \varphi }}\sigma_{{\text{r}}} - \frac{2C\cos \varphi }{{1 - \sin \varphi }} = 0$$

16 $$\frac{{{\text{d}}\sigma_{{\text{r}}} }}{{\sigma_{{\text{r}}} + C\cot \varphi }} = \frac{2\sin \varphi }{{1 - \sin \varphi }} \cdot \frac{{{\text{d}}r}}{r}$$

Available:

17 $$\ln (\sigma_{{\text{r}}} + C\cot \varphi ) = \frac{2\sin \varphi }{{1 - \sin \varphi }}\ln r + \ln A$$

18 $$\sigma_{{\text{r}}} + C\cot \varphi = Ar^{{\frac{2\sin \varphi }{{1 - \sin \varphi }}}}$$

where, *A* is the integral constant. According to *r* = *r*_1_, there is *σ*_r_ = 0, then:

19 $$A = \frac{C\cot \varphi }{{r_{1}^{{\frac{2\sin \varphi }{{1 - \sin \varphi }}}} }}$$

The radial stress and tangential stress of surrounding rock of unloading hole in limit equilibrium zone can be expressed as follows:

20 $$\sigma_{{\text{r}}} = C\cot \varphi \left[ {\left( {\frac{r}{{r_{1} }}} \right)^{{\frac{2\sin \varphi }{{1 - \sin \varphi }}}} - 1} \right]$$

21 $$\sigma_{{\text{t}}} = C\cot \varphi \left[ {\frac{1 + \sin \varphi }{{1 - \sin \varphi }}\left( {\frac{r}{{r_{1} }}} \right)^{{\frac{2\sin \varphi }{{1 - \sin \varphi }}}} - 1} \right]$$

**Figure 8:**
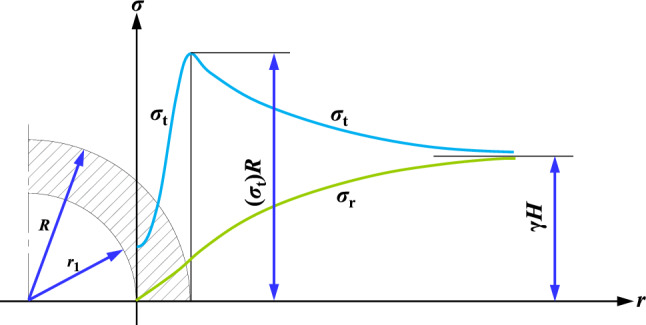
Tangential stress and radial stress distribution of lateral rock of circular hole.

Accordingly, the stress distribution in both ribs of circular hole to surrounding rock is shown in Fig. 8.

Assuming that the in-situ stress of surrounding rock of unloading hole is the hydrostatic field, that is, *λ* = 1. At the boundary *R* of limit equilibrium zone, equation $\sigma_{{\text{r}}} + \sigma_{{\text{t}}} = 2\sigma_{{1}}$ is followed. If $\sigma_{{1}} = \lambda H$, then:

22 $$C\cot \varphi \left[ {\frac{1 + \sin \varphi }{{1 - \sin \varphi }}\left( {\frac{R}{{r_{1} }}} \right)^{{\frac{2\sin \varphi }{{1 - \sin \varphi }}}} - 1} \right] + C\cot \varphi \left[ {\left( {\frac{R}{{r_{1} }}} \right)^{{\frac{2\sin \varphi }{{1 - \sin \varphi }}}} - 1} \right] = 2\gamma H$$

Namely:

23 $$R = r_{1} \left[ {\frac{(\gamma H + C\cot \varphi )(1 - \sin \varphi )}{{C\cot \varphi }}} \right]^{{\frac{1 - \sin \varphi }{{2\sin \varphi }}}}$$

According to the coal parameters and its internal unloading-hole around coal roadway of Dongpang Mine: unloading-hole radius *r*_1_ = 0.5 m, coal bulk density *γ* = 25 kN/m^3^, coal seam burial depth *H* = 660 m, coal cohesion *C* = 0.8 MPa, internal friction angle *φ* = 18°. Substituting Eq. (23) above, the limit equilibrium zone range *R* = 3.21 m for surrounding rock of internal unloading hole is obtained, so the actual plastic zone distance *r*_2_ = 2.71 m after excluding the radius of unloading hole.

### Basic composition and micromorphology of high-water material

**Figure 9:**
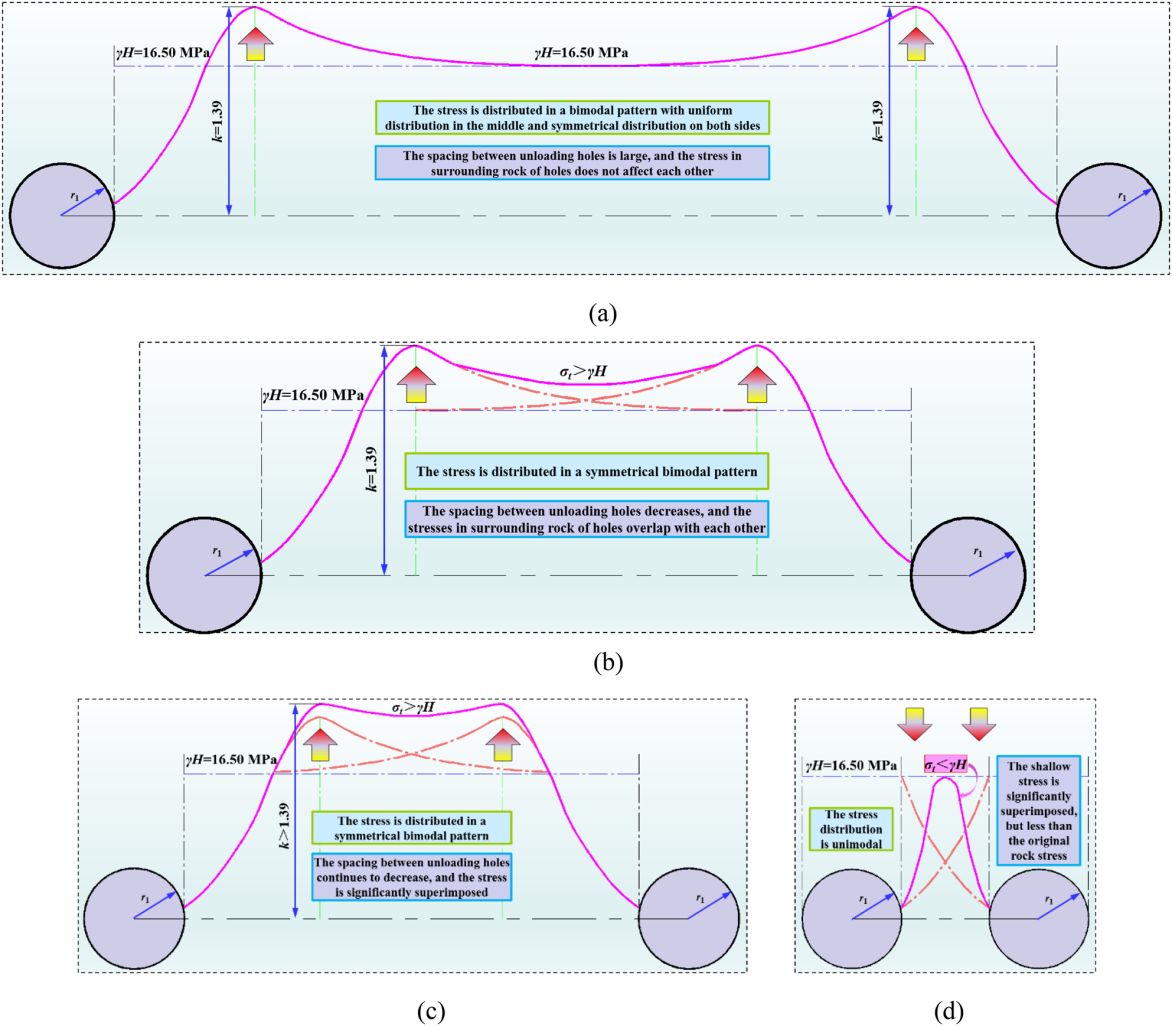
Abutment pressure distribution of surrounding rock under different unloading-hole spaces. (**a**) Unloading-hole spacing is large (≥ 13.08 m), (**b**) 13.08 m > Unloading-hole spacing ≥ 8.46 m, (**c**) 8.46 m > Unloading-hole spacing ≥ 4.25 m, (**d**) Unloading-hole spacing < 4.25 m.

The segmented expression of tangential stress in lateral surrounding rock of internal unloading-hole of two ribs in coal roadway is: (1) the tangential stress in limit equilibrium zone in shallow part of unloading hole is shown in Eq. (21); (2) the tangential stress of surrounding rock in deep elastic zone is shown in Eq. (12). The Matlab software is used to obtain tangential stress of lateral surrounding rock at different position r and different spacing of unloading hole, as shown in Fig. 9.

From Fig. 9, it shows that: (1) when internal unloading-hole spacing is too large (≥ 13.08 m), the surrounding rock load at central position between unloading holes is approximately a uniform distribution of in-situ stress and unloading effect is not ideal. (2) when internal unloading-hole spacing is large (13.08 m > spacing ≥ 4.25 m), the abutment pressure of surrounding rock located at central position between unloading holes is superimposed to a certain extent, and the stress in the middle is greater than the in-situ stress, which does not achieve effective pressure relief of unloading-hole surrounding rock. (3) as internal unloading-hole spacing is less than 4.25 m, there is a single-peak distribution of abutment pressure between adjacent unloading holes, and the superimposed abutment pressure between unloading holes is less than the in-situ stress, thus achieving effective pressure relief of coal roadway surrounding-rock and internal unloading holes. At the same time, based on the progress of internal excavation during on-site engineering construction, it has been comprehensively determined that the reasonable spacing between internal unloading-holes should be less than 4.25 m and should not be too small.

### Reasonable internal unloading-hole spacing

In summary, it has been found that when the spacing between internal unloading-holes is greater than 5.0 m by numerical simulation, there is a clear peak-stress area near unloading holes, and the large concentrated stress makes unloading-hole surrounding-rock not fully depressurized. As unloading-hole spacing is 2 ~ 4 m, the surrounding-rock is in a low stress environment, which can achieve marvelous pressure relief effect. Meanwhile, combined with distribution laws of lateral abutment pressure in surrounding rock of unloading circular-hole obtained by theoretical calculation, it is concluded that reasonable internal unloading-hole spacing in two ribs of coal roadway should be less than 4.25 m. The reasonable internal unloading-hole spacing is determined to be 4.0 m combined with on-site construction and comprehensive economic benefits of the coal mine.

## Engineering test

Based on determining reasonable internal unloading parameters, a rock-beam mechanical model is constructed under fixed support at both ends of coal roadway roof. The stability of coal roadway surrounding-rock under different support forms is solved, and the important role of anchor cable beam-truss in controlling surrounding rock deformation is elucidated. A coupling control technology of anchoring and unloading of coal roadway is proposed, and engineering tests are conducted on the stability control of roadway surrounding-rock in Dongpang Mine to verify the rationality of coupling control technology of anchoring and unloading and parameters.

### Analysis of stress and bending moment of roadway roof under different supports

In order to accurately construct the mechanical model of coal roadway roof-beam with fixed supports at both ends, the following assumptions should be made: (1) the roof is a beam structure model with fixed supports at both ends; (2) the roof is the homogeneous rock mass bearing dead weight; (3) the roof strata are continuous materials without gaps; (4) the roof has isotropic characteristic along different directions.

The roof beam with fixed support at both ends belongs to statically indeterminate structure problem, which can be solved by force method or displacement method in structural mechanics^38^. Among them, the displacement method takes the nodal displacement of model structure as an unknown quantity to solve the model stress state. The classical equation of displacement method is as follows:

24 $$\left\{ \begin{gathered} r_{11} Z_{1} + r_{12} Z_{2} { + } \cdot \cdot \cdot { + }r_{{{\text{1n}}}} Z_{{\text{n}}} { + }R_{{{\text{1P}}}} { = 0} \hfill \\ \, r_{21} Z_{1} + r_{22} Z_{2} { + } \cdot \cdot \cdot { + }r_{{{\text{2n}}}} Z_{{\text{n}}} { + }R_{{{\text{2P}}}} { = 0} \hfill \\ \, \cdot \cdot \cdot \cdot \cdot \cdot \, \hfill \\ r_{{{\text{n}}1}} Z_{1} + r_{{{\text{n}}2}} Z_{2} { + } \cdot \cdot \cdot { + }r_{{{\text{nn}}}} Z_{{\text{n}}} { + }R_{{{\text{nP}}}} { = 0} \hfill \\ \end{gathered} \right.$$

where, *Z*_i_ and direction represent the key displacement and direction of original structure; *r*_ij_ is the additional constraint reaction in *Z*_i_ direction caused by the element displacement *Z*_j_ = 1, also known as stiffness coefficient; *R*_iP_ is the additional constraint reaction in *Z*_i_ direction caused by load, also known as free term.

**Figure 10:**
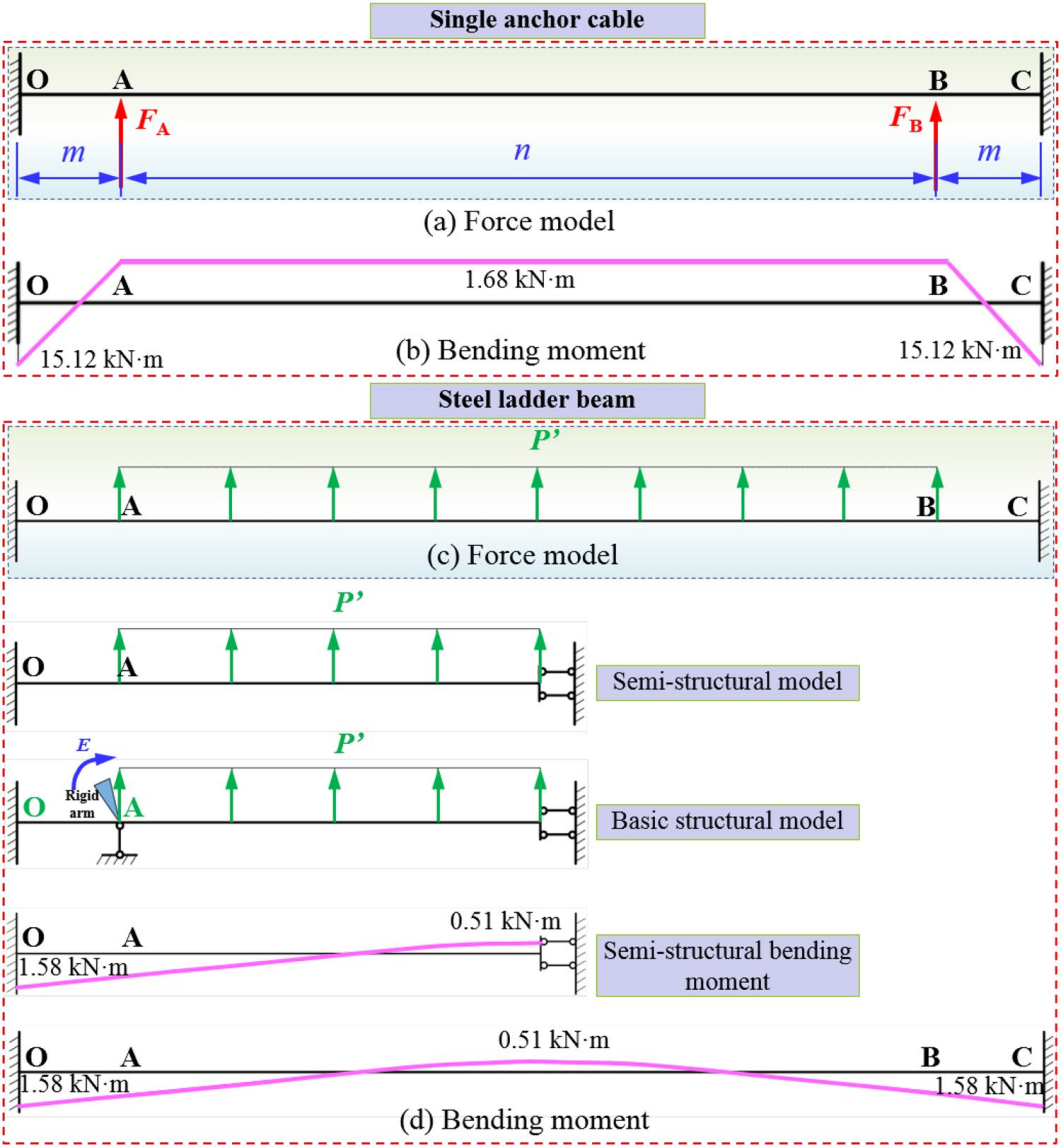
Force and bending moment of single anchor cables and steel ladder beam in roadway roof.

According to the calculation expression of bending moment at the end of the statically indeterminate bar of constant section by displacement method, the stress and bending moment of coal roadway roof-beam under different supports are obtained. The stress and bending moment of roof rock-beam supported by single anchor cables and reinforced ladder beam are shown in Fig. 10.

**Figure 11:**
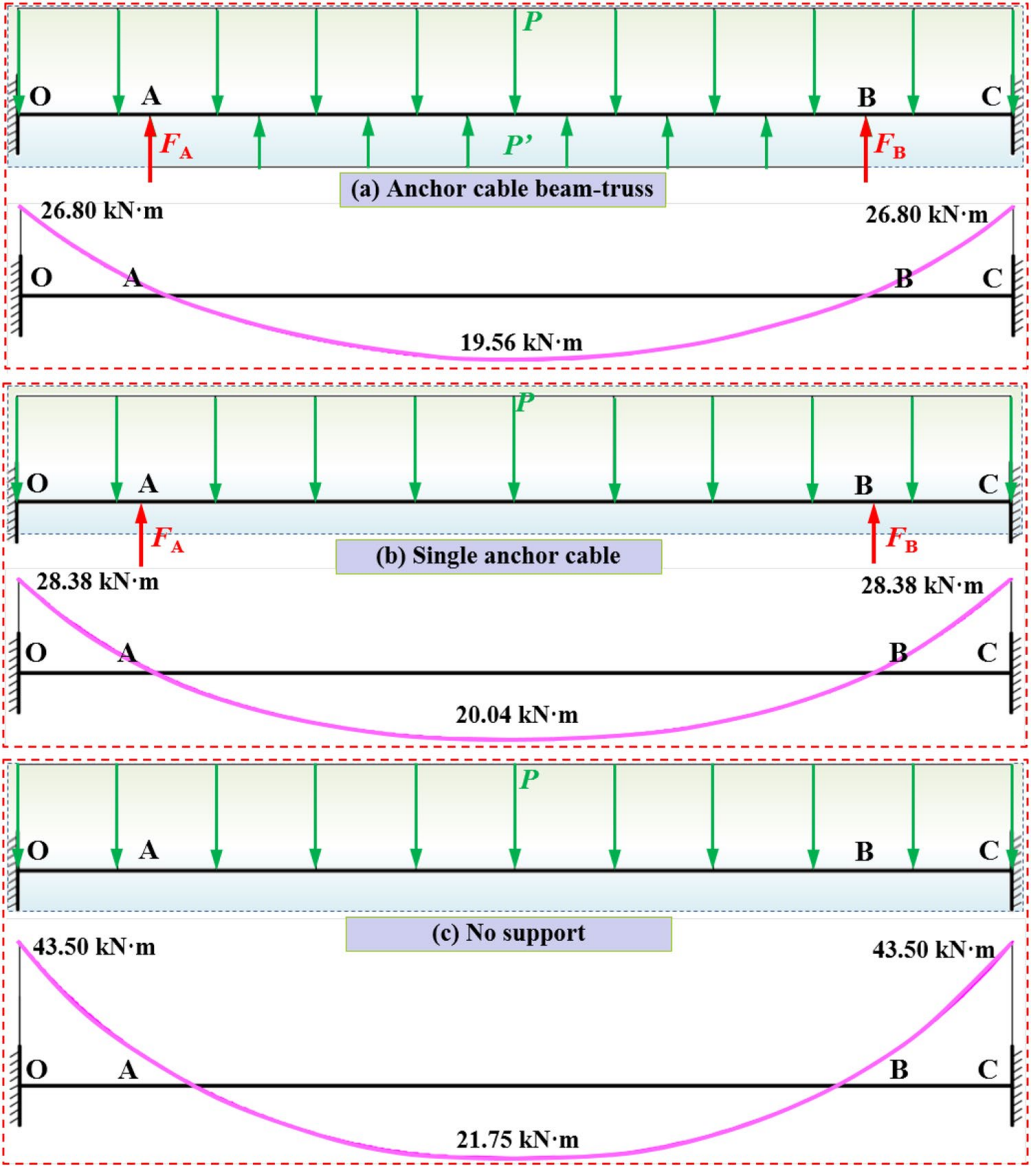
Force and bending moment of roadway roof with different supports under fixed support at both ends.

The support system of anchor cable beam-truss is mainly composed of anchor cables, double steel ladder beams and square tray. A comparison diagram of stress and bending moment of roof rock-beam in coal roadway under the conditions of anchor cable beam-truss, single anchor cables and no support is obtained, as shown in Fig. 11. Under the condition of fixed support at both ends of coal roadway roof with no support, the maximum bending moment is located on both ribs of fixed side section, the maximum bending moment is 43.50 kN·m, and the middle section bending moment of the roof is 21.75 kN·m. The bending moment of the roof is greater than the other two types of support. When single anchor cables are adopted on both ribs of the roof, the maximum bending moment at fixed side support of the roof is 28.38 kN·m, and the bending moment at middle roof section is 20.04 kN·m. The bending moment is reduced to a certain extent compared with no support. When the roof adopts the anchor cable beam-truss support, the roof bending moment on both ribs of fixed support and the central are reduced, the maximum bending moment at fixed support on both ribs is reduced to 26.80 kN·m, and the bending moment of rock-beam in the middle roof is reduced to 19.56 kN·m. Both ribs of roof support and bending moment at the central cross section are significantly reduced, which is conducive to maintaining the roof stability of coal roadway, and verifies the important role of anchor cable beam-truss system in controlling the roof stability.

**Figure 12:**
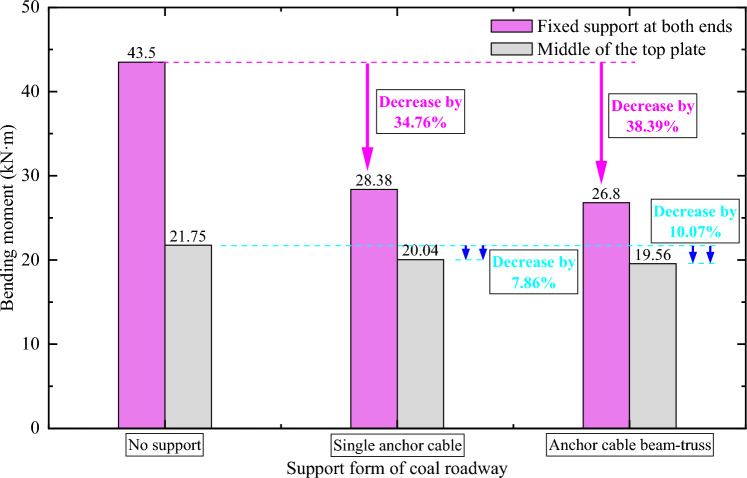
Bending moment for different supports of roadway roof under fixed supports at both ends.

In order to further analyze the changes of maximum bending moments at different positions of coal roadway roof under different supports, the maximum bending moments of rock beam at both ends of roof and central cross section under different supports are shown in Fig. 12.

It can be seen from Fig. 12 that when roadway roof is supported by single anchor cables at both ends, the maximum bending moments at both ends of roof support and the central section decrease by 34.76% and 7.86%, respectively compared with no support. The maximum bending moments at these positions decrease by 38.39% and 10.07%, respectively as anchor cable beam-truss structure is adopted. Therefore, the anchor cable beam-truss structure significantly reduces the maximum bending moment of coal roadway roof. The structure has a more significant control effect on surrounding rock compared with single anchor cables, improving the stress state of roadway surrounding-rock and promoting the stability of coal roadway.

### Coupling control parameters of anchoring and unloading for coal roadway

**Figure 13:**
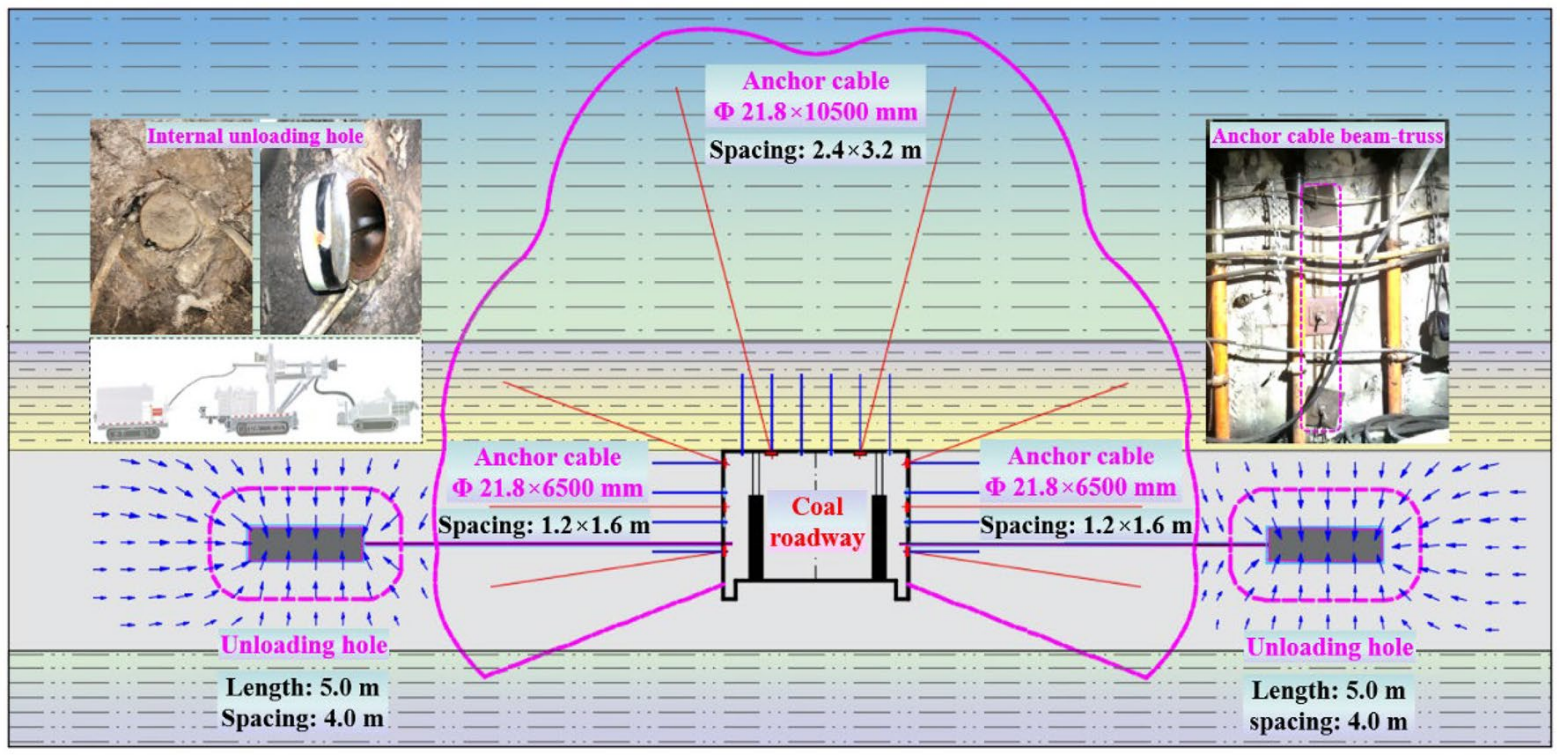
Coupling control parameters of anchoring and unloading for coal roadway.

Two grouting anchor cables of Φ21.8 × 10,500 mm are added to each row of coal roadway roof for strengthening support, and the row spacing is 2.40 m and 3.20 m. The grouting pipe extends into the anchor cable hole and uses chemical slurry to modify surrounding rock, with grouting pressure of 3.0 MPa. After chemical slurry has solidified, the anchor cables should be tensioned in a timely manner, and the pre tightening force of anchor cables on roadway roof should not be less than 130 kN. Three grouting anchor cables of Φ21.8 × 6500 mm are added to each row of two ribs of coal roadway, and the row spacing is 1.20 m and 1.60 m. When anchor cables are stabilized to the bottom of anchor cable hole, the grouting pipe is extended to anchor cable hole and surrounding rock is grouting modified with chemical material. The grouting pressure is 3.0 MPa, and the pre-tightening force of anchor cables in two ribs of coal roadway is not less than 100 kN. Meanwhile, according to the engineering geology of coal roadway in No.12 mining area of Dongpang Mine and the working attributes of crawler hydraulic drill, the reasonable unloading parameters of coal roadway are comprehensively determined as follows: The unloading holes are approximately cylindrical holes with a diameter of 1.0 m and a length of 5.0 m. The height of unloading hole is not less than 1.20 m from the bottom. The unloading holes of coal roadway are 10.0 m from roadway wall, and internal unloading-holes spacing is 4.0 m. In order to resist large deformation and failure of roadway surrounding-rock, comprehensive control technologies such as supporting, grouting modification and internal unloading are strengthened. The technical parameters and construction of coupling control of coal roadway in No.12 mining area of Dongpang Mine are shown in Fig. 13.

### Observation results and analysis of roadway mine-pressure

#### Displacement and roof separation of coal roadway

**Figure 14:**
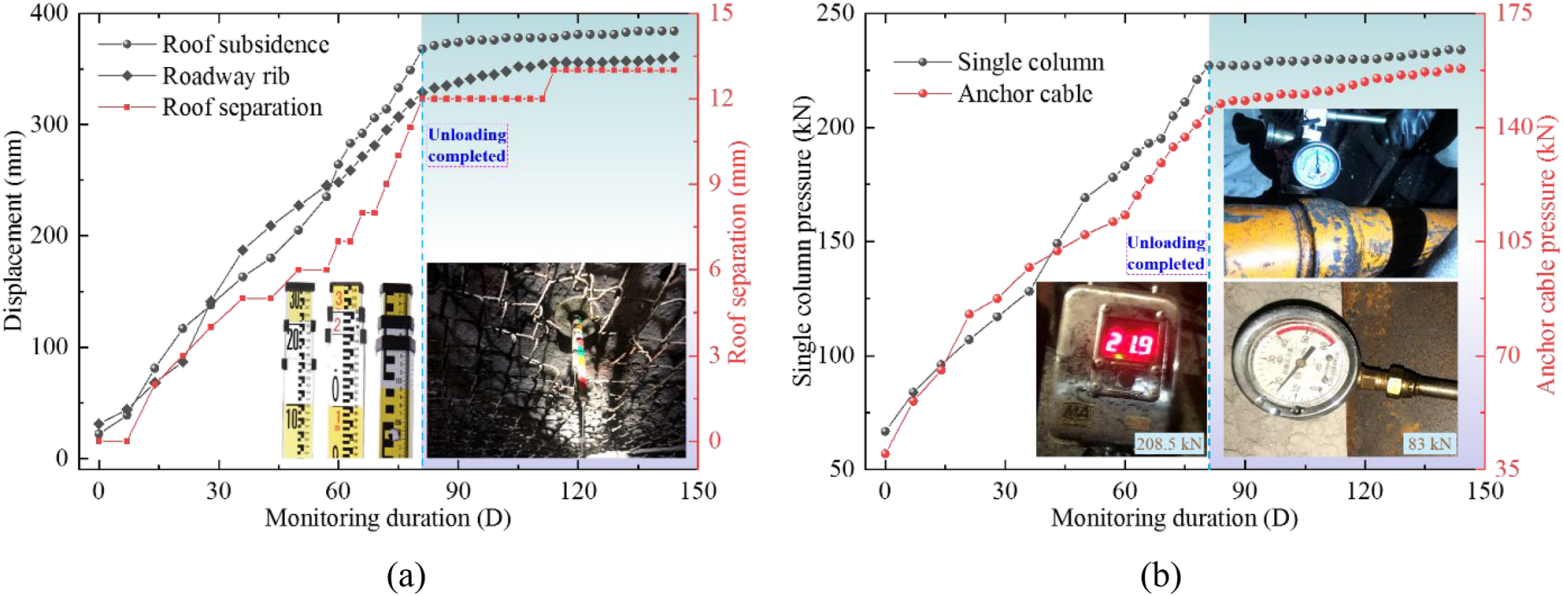
Observation results of mine pressure in coal roadway. (**a**) Displacement and roof separation, (**b**) Pressure of single columns and anchor cables.

The observation results of surrounding rock displacement and roof subsidence are shown in Fig. 14a. The roof separation and displacement of coal roadway tend to be stable after unloading. The maximum displacement is not more than 390 mm, and the maximum roof separation is not more than 13 mm. Therefore, the coupling control technology of anchoring and unloading can significantly control large deformation of surrounding rock in deep coal roadway.

#### Pressure of single columns and anchor cables

Figure 14b shows the pressure curves of single columns and anchor cables in coal roadway before and after unloading. The pressure continues to increase before the coupling control technology of anchoring and unloading, which is easy to cause instability and failure of coal roadway. The pressure growth rate of single columns and anchor cables in coal roadway surrounding-rock slows down obviously after adopting the coupling control technology, and finally tends to be stable. The pressure of single column is no more than 235 kN and the pressure of anchor cable is no more than 160 kN after stability, which is approximately 52.29% of the anchor breaking load. The coupling technology improves the stress state of coal roadway surrounding-rock, ensures the safety and stability of coal roadway during its service period, and verifies the applicability of coupling control technology of anchoring and unloading to resist large deformation of deep coal roadway surrounding-rock.

## Conclusions

1. The underground test results show that the minimum principal stress of roadway surrounding-rock in Dongpang Mine has exceeded the compressive strength of coal, up to 3.25 times of coal strength, and the maximum principal stress is 6.30 times of uniaxial compressive strength of coal, which indicates that coal roadway is prone to plastination and failure under deep high-stress state.
2. The joint failure mechanism of coal mass in the deep area migrates into coal roadway and acts on the anchoring support system is analyzed and expounded. It is concluded that conventional strengthening support and grouting modification techniques are no longer suitable for roadway surrounding-rock control in integrated extrusion deformation of coal mass structure. The improvement strategy named coupling control technology of anchoring and unloading for coal roadway is proposed.
3. Based on the distribution laws of surrounding rock stress under different unloading-hole spacing, it is concluded that surrounding rock stress will be superimposed as unloading-hole spacing is more than 4.25 m, and the roadway surrounding-rock has no obvious unloading effect. As unloading-hole spacing is less than 4.25 m, the maximum abutment pressure does not exceed the in-situ stress. The reasonable spacing of internal unloading-holes is determined to be 4.0 m combined with numerical simulation.
4. It is obtained that anchor cable beam-truss support is beneficial to maintain the stability of coal roadway by constructing a mechanical model of roof beam. The technical parameters named grouting anchor cable beam-truss support and internal-cavitation unloading for coal roadway are put forward. The experimental results of Dongpang Mine show that coupling control technology can significantly improve the stress state of roadway surrounding-rock, which verifies that coupling control technology of anchoring and unloading plays an important role in resisting large deformation of deep coal-roadway.

## Data Availability

All data and models or used during the study appear in the submitted article.
